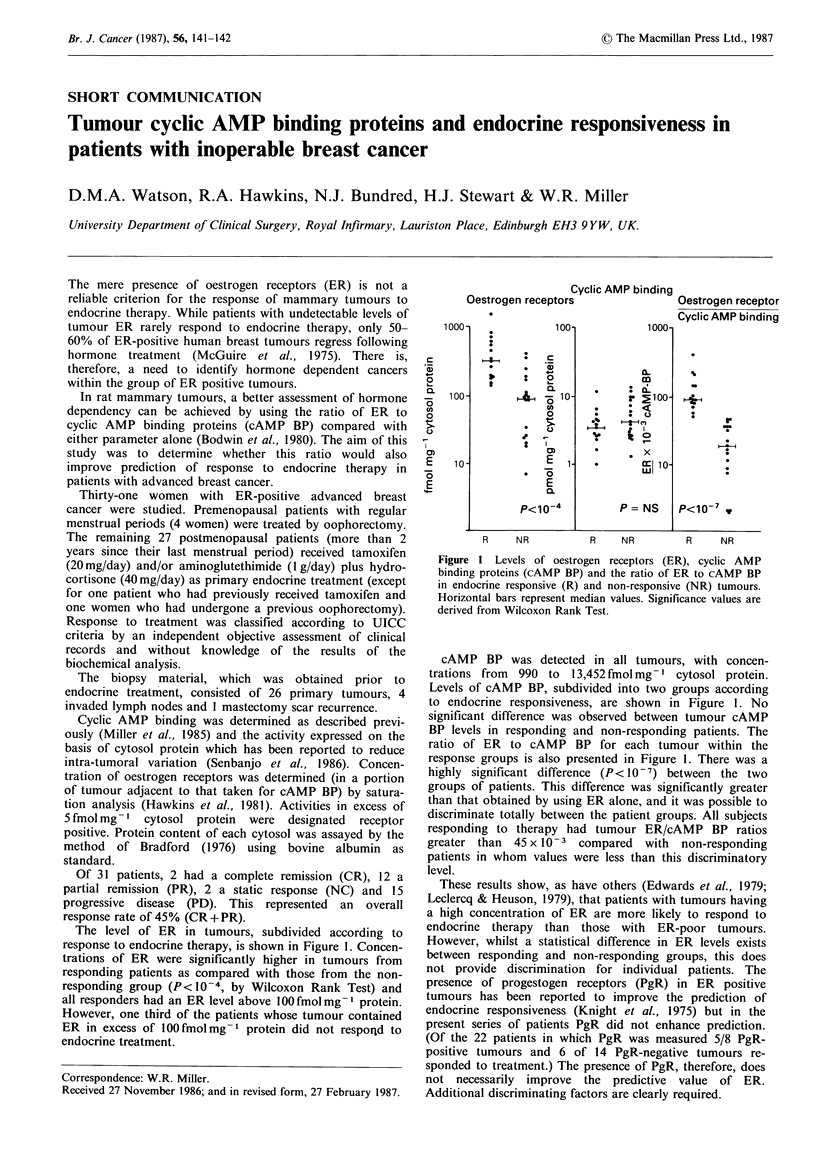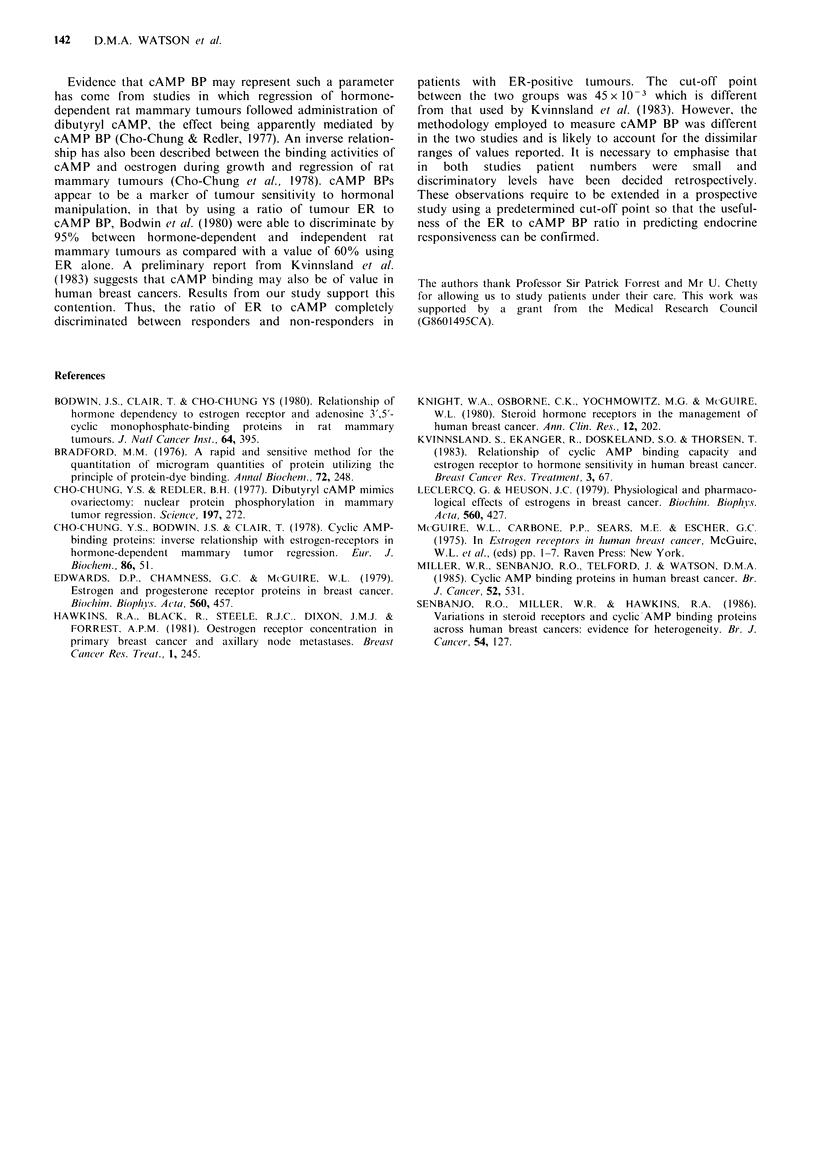# Tumour cyclic AMP binding proteins and endocrine responsiveness in patients with inoperable breast cancer.

**DOI:** 10.1038/bjc.1987.172

**Published:** 1987-08

**Authors:** D. M. Watson, R. A. Hawkins, N. J. Bundred, H. J. Stewart, W. R. Miller

**Affiliations:** University Department of Clinical Surgery, Royal Infirmary, Edinburgh, UK.


*Br. J. Cancer* (1987), **56**, 141–142 © The Macmillan Press Ltd., 1987

SHORT COMMUNICATION

### Tumour cyclic AMP binding proteins and endocrine responsiveness in patients with inoperable breast cancer

D.M.A. Watson, R.A. Hawkins, N.J. Bundred, H.J. Stewart & W.R. Miller

*University Department of Clinical Surgery, Royal Infirmary, Lauriston Place, Edinburgh EH3 9YW, UK.*

The mere presence of oestrogen receptors (ER) is not a reliable criterion for the response of mammary tumours to endocrine therapy. While patients with undetectable levels of tumour ER rarely respond to endocrine therapy, only 50–60% of ER-positive human breast tumours regress following hormone treatment (McGuire *et al.*, 1975). There is, therefore, a need to identify hormone dependent cancers within the group of ER positive tumours.

In rat mammary tumours, a better assessment of hormone dependency can be achieved by using the ratio of ER to cyclic AMP binding proteins (cAMP BP) compared with either parameter alone (Bodwin *et al.*, 1980). The aim of this study was to determine whether this ratio would also improve prediction of response to endocrine therapy in patients with advanced breast cancer.

Thirty-one women with ER-positive advanced breast cancer were studied. Premenopausal patients with regular menstrual periods (4 women) were treated by oophorectomy. The remaining 27 postmenopausal patients (more than 2 years since their last menstrual period) received tamoxifen (20 mg/day) and/or aminoglutethimide (1 g/day) plus hydrocortisone (40 mg/day) as primary endocrine treatment (except for one patient who had previously received tamoxifen and one women who had undergone a previous oophorectomy). Response to treatment was classified according to UICC criteria by an independent objective assessment of clinical records and without knowledge of the results of the biochemical analysis.

The biopsy material, which was obtained prior to endocrine treatment, consisted of 26 primary tumours, 4 invaded lymph nodes and 1 mastectomy scar recurrence.

Cyclic AMP binding was determined as described previously (Miller *et al.*, 1985) and the activity expressed on the basis of cytosol protein which has been reported to reduce intra-tumoral variation (Senbanjo *et al.*, 1986). Concentration of oestrogen receptors was determined (in a portion of tumour adjacent to that taken for cAMP BP) by saturation analysis (Hawkins *et al.*, 1981). Activities in excess of 5 fmol mg$^{-1}$ cytosol protein were designated receptor positive. Protein content of each cytosol was assayed by the method of Bradford (1976) using bovine albumin as standard.

Of 31 patients, 2 had a complete remission (CR), 12 a partial remission (PR), 2 a static response (NC) and 15 progressive disease (PD). This represented an overall response rate of 45% (CR + PR).

The level of ER in tumours, subdivided according to response to endocrine therapy, is shown in Figure 1. Concentrations of ER were significantly higher in tumours from responding patients as compared with those from the non-responding group ($P<10^{-4}$, by Wilcoxon Rank Test) and all responders had an ER level above 100 fmol mg$^{-1}$ protein. However, one third of the patients whose tumour contained ER in excess of 100 fmol mg$^{-1}$ protein did not respond to endocrine treatment.

Correspondence: W.R. Miller.
Received 27 November 1986; and in revised form, 27 February 1987.

**Figure 1** Levels of oestrogen receptors (ER), cyclic AMP binding proteins (cAMP BP) and the ratio of ER to cAMP BP in endocrine responsive (R) and non-responsive (NR) tumours. Horizontal bars represent median values. Significance values are derived from Wilcoxon Rank Test.

cAMP BP was detected in all tumours, with concentrations from 990 to 13,452 fmol mg$^{-1}$ cytosol protein. Levels of cAMP BP, subdivided into two groups according to endocrine responsiveness, are shown in Figure 1. No significant difference was observed between tumour cAMP BP levels in responding and non-responding patients. The ratio of ER to cAMP BP for each tumour within the response groups is also presented in Figure 1. There was a highly significant difference ($P<10^{-7}$) between the two groups of patients. This difference was significantly greater than that obtained by using ER alone, and it was possible to discriminate totally between the patient groups. All subjects responding to therapy had tumour ER/cAMP BP ratios greater than $45\times10^{-3}$ compared with non-responding patients in whom values were less than this discriminatory level.

These results show, as have others (Edwards *et al.*, 1979; Leclercq & Heuson, 1979), that patients with tumours having a high concentration of ER are more likely to respond to endocrine therapy than those with ER-poor tumours. However, whilst a statistical difference in ER levels exists between responding and non-responding groups, this does not provide discrimination for individual patients. The presence of progestogen receptors (PgR) in ER positive tumours has been reported to improve the prediction of endocrine responsiveness (Knight *et al.*, 1975) but in the present series of patients PgR did not enhance prediction. (Of the 22 patients in which PgR was measured 5/8 PgR-positive tumours and 6 of 14 PgR-negative tumours responded to treatment.) The presence of PgR, therefore, does not necessarily improve the predictive value of ER. Additional discriminating factors are clearly required.

Evidence that cAMP BP may represent such a parameter has come from studies in which regression of hormone-dependent rat mammary tumours followed administration of dibutyryl cAMP, the effect being apparently mediated by cAMP BP (Cho-Chung & Redler, 1977). An inverse relationship has also been described between the binding activities of cAMP and oestrogen during growth and regression of rat mammary tumours (Cho-Chung *et al.*, 1978). cAMP BPs appear to be a marker of tumour sensitivity to hormonal manipulation, in that by using a ratio of tumour ER to cAMP BP, Bodwin *et al.* (1980) were able to discriminate by 95% between hormone-dependent and independent rat mammary tumours as compared with a value of 60% using ER alone. A preliminary report from Kvinnsland *et al.* (1983) suggests that cAMP binding may also be of value in human breast cancers. Results from our study support this contention. Thus, the ratio of ER to cAMP completely discriminated between responders and non-responders in patients with ER-positive tumours. The cut-off point between the two groups was $45 \times 10^{-3}$ which is different from that used by Kvinnsland *et al.* (1983). However, the methodology employed to measure cAMP BP was different in the two studies and is likely to account for the dissimilar ranges of values reported. It is necessary to emphasise that in both studies patient numbers were small and discriminatory levels have been decided retrospectively. These observations require to be extended in a prospective study using a predetermined cut-off point so that the usefulness of the ER to cAMP BP ratio in predicting endocrine responsiveness can be confirmed.

The authors thank Professor Sir Patrick Forrest and Mr U. Chetty for allowing us to study patients under their care. This work was supported by a grant from the Medical Research Council (G8601495CA).

### References

BODWIN, J.S., CLAIR, T. & CHO-CHUNG YS (1980). Relationship of hormone dependency to estrogen receptor and adenosine 3′,5′-cyclic monophosphate-binding proteins in rat mammary tumours. *J. Natl Cancer Inst.*, **64**, 395.

BRADFORD, M.M. (1976). A rapid and sensitive method for the quantitation of microgram quantities of protein utilizing the principle of protein-dye binding. *Annal Biochem.*, **72**, 248.

CHO-CHUNG, Y.S. & REDLER, B.H. (1977). Dibutyryl cAMP mimics ovariectomy: nuclear protein phosphorylation in mammary tumor regression. *Science*, **197**, 272.

CHO-CHUNG, Y.S., BODWIN, J.S. & CLAIR, T. (1978). Cyclic AMP-binding proteins: inverse relationship with estrogen-receptors in hormone-dependent mammary tumor regression. *Eur. J. Biochem.*, **86**, 51.

EDWARDS, D.P., CHAMNESS, G.C. & McGUIRE, W.L. (1979). Estrogen and progesterone receptor proteins in breast cancer. *Biochim. Biophys. Acta*, **560**, 457.

HAWKINS, R.A., BLACK, R., STEELE, R.J.C., DIXON, J.M.J. & FORREST, A.P.M. (1981). Oestrogen receptor concentration in primary breast cancer and axillary node metastases. *Breast Cancer Res. Treat.*, **1**, 245.

KNIGHT, W.A., OSBORNE, C.K., YOCHMOWITZ, M.G. & McGUIRE, W.L. (1980). Steroid hormone receptors in the management of human breast cancer. *Ann. Clin. Res.*, **12**, 202.

KVINNSLAND, S., EKANGER, R., DOSKELAND, S.O. & THORSEN, T. (1983). Relationship of cyclic AMP binding capacity and estrogen receptor to hormone sensitivity in human breast cancer. *Breast Cancer Res. Treatment*, **3**, 67.

LECLERCQ, G. & HEUSON, J.C. (1979). Physiological and pharmacological effects of estrogens in breast cancer. *Biochim. Biophys. Acta*, **560**, 427.

McGUIRE, W.L., CARBONE, P.P., SEARS, M.E. & ESCHER, G.C. (1975). In *Estrogen receptors in human breast cancer*, McGuire, W.L. *et al.*, (eds) pp. 1–7. Raven Press: New York.

MILLER, W.R., SENBANJO, R.O., TELFORD, J. & WATSON, D.M.A. (1985). Cyclic AMP binding proteins in human breast cancer. *Br. J. Cancer*, **52**, 531.

SENBANJO, R.O., MILLER, W.R. & HAWKINS, R.A. (1986). Variations in steroid receptors and cyclic AMP binding proteins across human breast cancers: evidence for heterogeneity. *Br. J. Cancer*, **54**, 127.